# Opening a Large Delivery Service Warehouse in the South Bronx: Impacts on Traffic, Air Pollution, and Noise

**DOI:** 10.3390/ijerph17093208

**Published:** 2020-05-05

**Authors:** Jenni A. Shearston, A. Mychal Johnson, Arce Domingo-Relloso, Marianthi-Anna Kioumourtzoglou, Diana Hernández, James Ross, Steven N. Chillrud, Markus Hilpert

**Affiliations:** 1 Department of Environmental Health Sciences, Mailman School of Public Health, Columbia University, New York, NY 10032, USA; js5431@cumc.columbia.edu (J.A.S.); ad3531@cumc.columbia.edu (A.D.-R.); mk3961@cumc.columbia.edu (M.-A.K.); 2 South Bronx Unite (Co-Founder), New York, NY 10454, USA; mychaljohnson@gmail.com; 3 Department of Sociomedical Sciences, Mailman School of Public Health, Columbia University, New York, NY 10032, USA; dh2494@cumc.columbia.edu; 4 Lamont-Doherty Earth Observatory of Columbia University, Palisades, NY 10964, USA; jross@ldeo.columbia.edu (J.R.); chilli@ldeo.columbia.edu (S.N.C.)

**Keywords:** traffic related air pollution, traffic flow, black carbon, noise, environmental justice, natural experiment

## Abstract

Mott Haven, a low-income neighborhood in New York City, suffers from increased air pollution and accommodates several industrial facilities and interstates. In 2018, a large delivery service warehouse opened. Our objectives are to characterize black carbon (BC), fine particulate matter (PM_2.5_), and noise in the community; model changes in traffic due to the facility opening; and estimate associated BC and noise changes. BC, PM_2.5_, and noise were measured at eight sites pre-opening, and traffic counted continuously at two sites (June 2017–May 2019). An interrupted time series model was used to determine facility-related changes in traffic. Post-opening changes in traffic-related BC/noise were estimated from regressions of BC/noise with traffic flow. Mean (SD) pre-warehouse measures of BC and PM_2.5_ were 1.33 µg/m^3^ (0.41) and 7.88 µg/m^3^ (1.24), respectively. At four sites, equivalent sound levels exceeded the EPA’s recommended 70 dBA limit. After the warehouse opening, traffic increased significantly, predominantly at night. At one site, the greatest change for trucks occurred 9PM-12AM: 31.7% (95%CI [23.4%, 40.6%]). Increased traffic translated into mean predicted increases of 0.003 µg/m^3^ (BC) and 0.06 dBA (noise). Though small, they negate the substantial decrease the community seeks. Our findings can help communities and policymakers better understand impacts of traffic-intensive facilities.

## 1. Introduction

Environmental justice concerns arise when vulnerable neighborhoods are overburdened with environmental exposures, including heavy traffic and industrial facilities, which can negatively impact air quality. The health effects of air pollution have been shown to be differentially harmful, such that worse outcomes are observed for populations with lower socio-economic status (SES) and ethnic and racial minorities [1,2,3]. This evidence suggests that local governments should preferentially target these neighborhoods for pollution reduction, but unfortunately this does not always happen, e.g., because of political or economic influences. The Mott Haven and Port Morris neighborhoods in New York City (NYC), comprised primarily of individuals with lower SES and who are largely ethnic and racial minorities [4], are an example of this occurrence. In 2018, an additional trucking-intensive facility was opened at the invitation of state and local government, potentially halting the NYC-wide trend of air pollution reduction [5,6] for this vulnerable and overburdened community. 

The Mott Haven and Port Morris neighborhoods experience higher than average air pollution, with an annual average fine particulate matter (particles with aerodynamic diameter ≤ 2.5 μm; PM_2.5_) level of 8.6 µg/m^3^, greater than both the Bronx borough wide average (7.8 µg/m^3^) and the NYC average (7.5 µg/m^3^) [4]. Traffic related pollution, including both air pollution and noise, is of particular concern, as multiple interstate highways run through the South Bronx, and approximately 20% of all preschool to 8th grade students attend a school within close proximity to a major highway [7]. Healthwise, Mott Haven has a very high incidence of child asthma emergency department visits, at 647 visits per 10,000 children aged 5 to 17, compared to the Bronx (410 visits) and NYC (223 visits) [4]. Other health concerns include elevated obesity, diabetes, and hypertension rates [4], which can be exacerbated by air pollution, and disturbances and health effects from traffic-related noise. In addition, Mott Haven has nearly double the rate of pedestrian injury hospitalizations than NYC as a whole, at 43 versus 23 hospitalizations per 100,000 people [4].

Consumer goods are increasingly purchased online and then delivered to consumers, where the last leg of delivery typically involves ground transportation via delivery trucks. These trucks take the goods from a distribution center to the customers, while larger trucks deliver the goods to the distribution center. Little is known about how this shift in commerce affects air quality and human health, or about how the opening of such a facility can impact a local community through traffic related air pollution, noise and congestion. Despite high air pollution and asthma rates, in 2012, an online grocery delivery service warehouse was promised more than $100 million in New York City and State subsidies to relocate its food distribution facility to the Mott Haven area of the South Bronx, before any public hearing on the matter [8].

The proposed move was controversial. While some elected officials favored the move, members of the local community, including the community organization South Bronx Unite (SBU), were concerned about additional truck traffic and associated adverse impacts on air pollution, pedestrian and bicyclist safety, and community health, as evidenced by discussion in the local newspaper [9,10]. The Mott Haven area already has multiple major sources of air pollution, including two large interstates, a large food distribution hub in nearby Hunts Point that handles food for the entirety of NYC, and two waste transfer stations, one of which receives municipal waste for the entire Bronx borough. The community argued that they could not shoulder additional truck traffic in their environmentally overburdened community, potentially resulting in further negative health consequences. They also argued that the assessment of the environmental impacts of the proposed facility should not have been based on an environmental impact statement (EIS) that (1) was 19 years old [11], (2) that failed to consider PM_2.5_ (since PM_2.5_ was not added to the U.S. National Ambient Air Quality Standards until 1997), and (3) that failed to take into account substantial increases in residential inhabitants to the area, including as a result two rezonings [12].

In response to community concerns, our goal is to quantify the impact of the opening of the online grocery delivery service warehouse on traffic related pollution, including traffic flow, air pollution, and noise pollution. The objectives of this study are to: (1) characterize levels of traffic pollution, including vehicle volume, BC levels, and noise levels, in a community already heavily affected by traffic; (2) model changes in traffic flow as a result of the opening of an online grocery delivery service warehouse in the neighborhood; and (3) estimate increases in traffic-related BC and noise due to increased traffic volume after the warehouse opening. Our study approach is original, because we collected traffic radar data before and after an intervention in order to assess associated impacts on diurnal traffic patterns, air pollution, and noise in a residential community. To the best of our knowledge, our study is the first one in which traffic increases due to the opening of a trucking-intensive operation in a low-income, highly populated neighborhood were measured.

## 2. Methods

### 2.1. Overview

**Figure 1:**
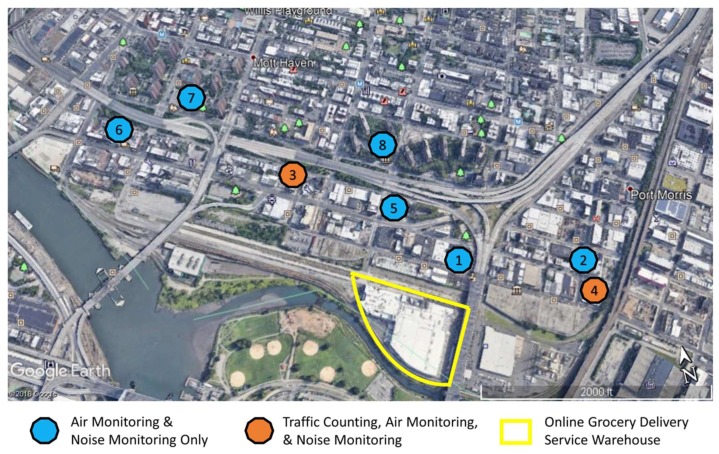
Study site, including locations used for traffic counting, air quality measures, and noise measures.

This study examines a natural experiment that occurred in the South Bronx region of NYC: the opening of a new online grocery delivery service warehouse. A map of the study site, including the locations of the warehouse and monitors for traffic counting, air quality measures, and noise, is shown in Figure 1. Construction of the exterior of the warehouse was completed in the Fall of 2016 or even earlier. The community expected the warehouse to start operating in 2017. Pre-warehouse opening measures were therefore collected for air quality and noise in 2017. The warehouse eventually opened in the summer of 2018, probably gradually ramping up its capacity. Post-warehouse opening measures were not collected, because modeling based on the traffic increase projected in the environmental assessment (EA) and the mobile-source contributions to levels of BC (a tracer for traffic-related air pollution) we determined from pre-opening data [13] indicated that the raw post-opening BC and PM levels would not be necessarily higher than the pre-opening ones due to variability in meteorological conditions and background pollution levels. Traffic counts were continuously collected throughout the study period, providing for pre- and post-warehouse opening measures. Changes in traffic flow were used to estimate changes in air quality and noise, based on the relationships between traffic and pollutants in the pre-opening period [13]. 

### 2.2. Monitoring Sites

Measurements were taken at eight monitoring sites, “Sites 1–8.” The number in the Site ID indicates the order in which sites were taken into operation. Table 1 summarizes selected characteristics of the sites. Outdoor air was sampled through windows of residential homes (Sites 2, 3, 5, 6) and businesses (Site 1) facing a street, from the rooftop of a warehouse (Site 4), and from the rooftops of two common-usage areas in New York City Housing Authority (NYCHA) housing (Sites 7–8). The study was approved by the Columbia University Institutional Review Board. Informed consent was obtained from study participants (Sites 1–6) while a license agreement was executed between Columbia University and NYCHA. All sites were identified by our community partner SBU. The eight sites differ in their horizontal distance to the nearest road, traffic volume of that road according to the New York State Department of Transportation [14], functional classification codes according to the New York State Department of Transportation [15], and elevation (Table 1).

At Sites 1–6, measurements were taken before the warehouse opened, from May to October 2017. At Sites 7 and 8, measurements were taken from July to October 2018. It is possible that the warehouse was already partially operational during that period of time. We would have preferred to make measurements at Sites 7 and 8 also in the summer of 2017; however, at the time we did not yet have permission from NYCHA to install the monitors.

Noise and air monitors were collocated at the study sites, unless otherwise specified. All air monitors sampled outdoor air with inlets roughly 2-3 feet from outside walls or rooftops even when monitors were located inside residences. The air and noise monitors for Site 1 were located on the second floor of a business building near a one-lane one-way street in a mixed-use area. A school and playground were located on the opposite side of the building. At Site 2, the devices were located on the first floor of a residential building located on a one-lane one-way street. Devices at Site 3 were placed on the third floor of a residential building located on a one-lane one-way street that for the most part receives traffic from an interstate off-ramp. The air monitor at Site 4 was placed on the rooftop of a warehouse located on a one-lane one-way street, while the noise monitor was attached to a light pole directly above the street. At Site 5, both monitors were again placed on the third floor of a residential building, this time located at an intersection in a mixed-use area. The devices at Site 6 were located on the second floor of a residential building at a two-way four-lane street in a mixed-use area. At Sites 7 and 8, devices were located on rooftops of common-use spaces of two large residential complexes owned by NYCHA. 

Sites 3 and 4 were selected to monitor traffic because they are vastly different from each other. Site 3 is an exit ramp from one of the interstates that run through Mott Haven, US Interstate I-87, and serves as a high traffic throughput location. Site 4 is a small one-way street and serves a much smaller traffic flow, chiefly as a route to the Harlem River Yards industrial area, of which the online grocery delivery warehouse is a part. The radar counters for both sites were mounted on streetlight poles and captured one-way traffic at each location. 

### 2.3. Air Quality, Traffic, and Noise Monitoring

#### 2.3.1. Air Quality 

At the eight study sites, we obtained integrated BC and PM_2.5_ concentrations of outdoor air using custom Columbia University sampling boxes, which contain two 7 L/min vacuum pumps (Medo, model VP0465), each controlled by a timer for exact on off control and a counter for elapsed run time. Flow rates between 1.0 and 2.0 L/min can be chosen through a needle valve. A different needle valve can be installed for 4 L/min. Boxes can run indefinitely on wall power but are typically used for 7–28 day deployments. At five of the eight sites (Sites 1–3, 5, 6), the sampling boxes were placed indoors with sampling lines passing through a window unit that fits in double hung windows [16]. At the other sites (Sites 4, 7, 8), the sampling boxes were placed outdoors on a rooftop inside a plastic storage container that had holes at the bottom for the sampling line and the electrical power cord. 

For all units, outdoor air was pumped at a constant flow rate of 1.5 L/min through a size-selective inlet with a 2.5 μm cut point (triplex cyclone by BGI) with particles collected onto 37-mm Teflon filters (Pall). For PM_2.5_ levels, filters were pre- and post-weighed on a microbalance after being equilibrated in a temperature-humidity controlled environment for at least 24 hours [17]. For BC, filter deposits were analyzed optically [18]. Elapsed time counters together with the time logs from the field technicians changing the filters allowed us to identify potential power outages, which would have biased inferred BC and PM_2.5_ levels.

Sites 1–6 were visited about every two weeks, whereas Sites 7–8 about every four weeks (due to lack of resources). Integrations were done for the time period between site visits, ranging from 12 to 20 days for Sites 1–6 and 26 to 28 days for Sites 7–8.

#### 2.3.2. Traffic

Traffic radar devices (Armadillo traffic counter, Houston Radar) were operational from June 1, 2017 to May 5, 2019 at Sites 3 and 4. These devices record time and date of the detection, speed, and class for each vehicle it observes. We define vehicle class as follows: large vehicles (length > 7 m) represent “trucks,” the combination of small (length < 4 m), medium (4m < length < 7 m), and large vehicles represent “total vehicles,” and “cars” represent small and medium vehicles (or total vehicles minus trucks). The radar devices’ event logs can be used to ascertain truck, vehicle, and car flow in units of count/time period. For more information about the traffic collection locations or radar devices, please see Hilpert et al. [13].

#### 2.3.3. Noise

Sound intensity levels were measured as a metric of noise. Measurements were taken with sound level loggers (Extech model 407760), because this model has been used in a previous study of noise levels in NYC [19]. The loggers allowed measuring equivalent sound levels at a sampling rate of either 50 ms, 500 ms, 1 s, 2 s, 5 s, 10 s, or 60 s. While we would have preferred to use the smallest rate of 50 ms in order to capture short duration but very loud and harmful sounds, we chose 10 s to allow the logger’s internal memory to store 15 days of sound level data, corresponding approximately to the period of time between visits of Sites 1–6. For comparison with EPA environmental noise level limits [20], A-weighted sound levels (dBA) were recorded. Sound-level meters were calibrated before field deployment at 94 dB using an Extech 407766 Sound Calibrator. Noise levels measured close to building facades were not corrected for the presence of facades (Sites 1, 2, 3, 5, and 6) as described in guidelines by the International Organization for Standardization [21,22] because these corrections are intended to estimate environmental noise levels away from buildings [22].

### 2.4. Statistical Analyses

For all statistical analyses, the warehouse opening date was conservatively estimated to be October 1, 2018, although it is possible that the warehouse opened before this time or gradually. If the warehouse did open before October 1, 2018, our choice would bias our results towards the null. We selected this conservative estimate to ensure that measures defined as occurring post-warehouse opening captured any traffic change after the facility had begun operation and did not represent a traffic change from construction work that might have possibly occurred within the facility. 

#### 2.4.1. Objective 1: Characterizing Traffic Pollution

Pre-warehouse opening air pollution levels were characterized through time-integrated BC and PM_2.5_ levels, which represent averages over the period of times between site visits ranging from 12 to 28 days. We note that for Sites 7 and 8, data collection of air measures ended on October 3, 2018, three days past our pre-warehouse opening designation. We interpret these averaging periods as pre-opening, however, because the majority of days in the sampling period occurred in the pre-opening time window. To allow for consistency with air pollution measures, for each site and each time period between site visits, an equivalent sound level was obtained from the 10-s resolution noise-level data *L_eq,_*_10s_ collected between the visits through $L_{eq}=10\;\log_{10}\left((1/N)\sum_{i=1}^{N}10^{L_{eq,10s}(t_{i})\;/\;10}\;\right)$ where *N* is the number of sound level samples taken [23]. The date range of the sound-level data often did not correspond exactly with the date range of the air pollution measures. This happened when the time between visits exceeded 15 days (due to memory limitations in the sound level loggers) or when the battery of the sound monitor was depleted before a site visit (78% of visits). For comparison with the EPA noise-level limit of 70 dbA, we calculated for each site the equivalent sound level for all days for which measurements were taken for 24 h, *L_eq,tot_*. Moreover, we stratified this time period by time of week (weekday/weekend), and time of day (day 7 AM–10 PM and night 10 PM–7 AM) and calculated equivalent sounds levels *L_eq,weekday_*, *L_eq,weekend_*, *L_eq,daytime_*, and *L_eq,night_* for these four subperiods.

Descriptive statistics of traffic counts, including means and standard deviations (SD), were calculated for eight three-hour time windows before and after the facility opened: midnight to 3 AM, 3 AM to 6 AM, 6 AM to 9 AM, 9 AM to noon, noon to 3 PM, 3 PM to 6 PM, 6 PM to 9 PM, and 9 PM to midnight. Comparing 3-h instead of daily flows has two advantages: first, traffic data for an entire day does not need to be discarded or interpolated if only a small data gap exists (only the three-hour window needs to be adjusted), e.g., due to download of data from the radar devices; second, this choice allows us to study diurnal changes in traffic and understand corresponding impacts on the community. In addition, we chose 3-h windows over 1-h windows because choice of the time window is to some extent arbitrary and hourly plots looked too busy.

#### 2.4.2. Objective 2: Model Changes in Traffic Flow

To assess potential changes in traffic flow due to the opening of the online grocery delivery service warehouse, we used an interrupted time series model (ITS) [24] for traffic radar data collected continuously throughout the study period (Sites 3 and 4). Warehouse opening was coded as a binary variable *X_t_*: value of 1 indicates warehouse open (October 1, 2018 and after), and value of 0 indicates warehouse not open (prior to October. 1, 2018). We calculated traffic flows (trucks or total vehicles per time period) at a three-hour temporal resolution corresponding to the time windows used for the descriptive traffic statistics.

One generalized linear model was created for each of the eight time windows and for each study site with continuous traffic radar data (Sites 3 and 4), for ease in interpretation. We used quasi-Poisson distributions because our outcome was over-dispersed count data (traffic flows). All models were adjusted for day of the week (DoW, categorical 7-level variable) and long-term and seasonal trends (LTST) using a harmonic term with two sine/cosine pairs and a 12-month period: $N_{t}=\beta_{0}+\beta_{1}t+\beta_{2}X_{t}+\beta_{3}\mathrm{DoW}+\beta_{4}\mathrm{LTST}$ where *N_t_* is a 3-hourly traffic count, $\beta_{0}$ is the baseline traffic flow at t = 0, $\beta_{1}$ is the change in traffic for the passage of an additional day (the pre-warehouse opening trend in traffic), $\beta_{2}$ is the change in traffic following the opening of the warehouse $X_{t}$ (the β of interest), $\beta_{3}$ is the change in traffic due to day of week, and $\beta_{4}$ is the change in traffic due to long-term and seasonal trends. We note that the harmonic terms we used to describe seasonal traffic changes (LTSD) were also used to model weekly noise levels of urban road traffic [25]. To compare our modeled average flows of vehicles and trucks due to the warehouse opening with the numbers presented in the EA form filed on behalf of the online grocery delivery service warehouse in connection with its NYC subsidy application [26], we used the models to estimate for Sites 3 and 4 the times series of segregated traffic flows from October 2018–May 2019 attributable to the warehouse opening. These time series were obtained by subtracting the traffic flow time series from the ITS model prediction with the facility being opened on October 1, 2018 from the time series predicted for the hypothetical case in which the facility did not open. For each 3-hour time window and each site, we then obtained the average change in traffic flow during the post-opening period (October 2018-May 2019) for weekends and weekdays separately, as this was the way it was estimated in the EA. In total 32 models were created; given the strong effect sizes and consistent results we observed, we thought this number was appropriate and did not require *p*-value adjustment [27]. All confidence intervals are provided. 

#### 2.4.3. Objective 3: Estimating Increases in BC and Noise

To examine how pre-warehouse opening traffic affected sound levels at Sites 3 and 4, we fitted regression models to the measured 15-min time series of the traffic flow and noise. Regressions were performed for sound intensity levels I because, for physical reasons, sound intensity levels rather than decibels scale linearly with traffic flows [28,29]. We fitted the following models to the measured data:

(1) $$I(t)/I_{0}=\lambda_{car}Q_{car}(t)+\lambda_{tr}Q_{tr}(t)+s(t)$$

and

(2) $$I(t)/I_{0}=\lambda_{tot}Q_{tot}(t)+s(t)$$

where *I* is the sound intensity level with units of W/m^2^ averaged over 15 min, *t* is time, *I*_0_ = 10^−12^ W/m^2^ is the threshold of hearing intensity level, *Q_car_*(*t*) is the car flow, *Q_tr_*(*t*) is the truck flow, and *Q_tot_*(*t*) is the total vehicle flow (cars and trucks). All flows were determined from the traffic radar event logs for 15-min observational windows like in the traffic-BC analysis performed by Hilpert et al. [13]. Therefore, all time-dependent variables in Equations (1) and (2) are defined for 15-min observational windows. The spline *s*(*t*) with three degrees of freedom accounts for potential very slow drifts of the sound-level monitors. The 15-min sound intensity level *I* is related to the 15-min equivalent sound levels through $L_{eq,15\min}=10\log_{10}(I_{15\min}/I_{0})$ where $L_{eq,15\min}$ can be calculated from the measured 10-sec sound levels $L_{eq,10s}$ [23].

We fit the first model given by Equation (1) because it uses the segregated traffic counts obtained by the traffic radar. We fit the second model given by Equation (2) for comparison to existing or future traffic-sound level data only including *Q_tot_*. Our models are either consistent with [30,31] or very similar to [32,33] other regression models for traffic-related sound levels. 

For the regressions, Gamma generalized linear models (GLMs) [34] with a logarithmic link function were used, because sound intensity levels *I*(*t*) were not normally distributed. As model residuals could not be expected to be normally distributed, we used the DHARMa package [35] to produce interpretable residual plots. To examine potential collinearity between the predictor variables, the car and truck flows *Q_car_*(*t*) and *Q_tr_*(*t*), we determined Pearson correlation coefficients between the time series of *Q_car_*(*t*) and *Q_tr_*(*t*).

The regression coefficients *λ_car_* and *λ_tr_* can be used to estimate changes in sound intensity level due to changes in segregated traffic flows through the linear terms of a Taylor series expansion of Equation (1): $\Delta I/I_{0}=\lambda_{car}\;\Delta Q_{car}+\lambda_{tr}\;\Delta Q_{tr}$ where Δ*I* is the change in sound intensity level due to changes in the flows of cars, Δ*Q_car_*, and of trucks, Δ*Q_tr_*. For the 15-min equivalent sound levels, a similar relationship exists:$\Delta L_{eq,15\min}=(\partial L_{eq,15\min}/\partial Q_{car})\;\Delta \;Q_{car}+(\partial L_{eq,15\min}/\partial Q_{tr})\;\Delta \;Q_{tr}$ where $\partial L_{eq,15\min}/\partial Q_{car}=10\lambda_{car}\;I_{0}/I$ and $\partial L_{eq,15\min}/\partial Q_{tr}=10\lambda_{tr}\;I_{0}/I$ represent slope coeffcients, which in contrast to the Taylor series for *I* depend on the sound level itself (through the denominator *I*). To get a sense of the general impacts of changes in traffic on equivalent sound levels $L_{eq,15\min}$, we approximated for each site *I* by its median *I*_50_ and report for each site $\partial L_{eq,15\min}/\partial Q_{car}=10\lambda_{car}\;I_{0}/I_{50}$ and $\partial L_{eq,15\min}/\partial Q_{tr}=10\lambda_{tr}\;I_{0}/I_{50}$ in units of dB/(100 h^−1^) which reflect the change in dBA for a change in traffic volume of 100 vehicles per hour. Similarly, we estimate changes in $L_{eq,15\min}$ due to changes in total vehicle flow through $dL_{eq,15\min}/dQ_{tot}=10\lambda_{tot}\;I_{0}/I_{50}$.

To examine how traffic associated with the opening of the online grocery store affected BC levels at Sites 3 and 4, we used regression coefficients from the BC-traffic analysis previously performed at Sites 3 and 4 [13]. That study examined how BC levels measured in real time with aethalometers depended on various measured traffic characteristics [13]. 

To estimate the increase in BC associated with the increased traffic from the opening of the warehouse, we multiplied the average change in traffic flow for each site (estimated as part of Objective 2, as described above) by the calculated BC coefficients from Hilpert et al. [13] to arrive at estimates of increases due to the traffic change from the facility, for Sites 3 and 4. Similarly, to estimate the increase in noise associated with the increased traffic flow, we multiplied the average change in traffic flow for each site by the noise coefficients appearing in Equation 1, for Sites 3 and 4. 

Analyses were conducted with MATLAB version R2017b and R version 3.5.1 [36]. The R packages tidyverse [37] and lubridate [38] were used for data management, and patchwork [39] was used for some plots. 

## 3. Results

### 3.1. Objective 1: Levels of Traffic Pollution

**Figure 2:**
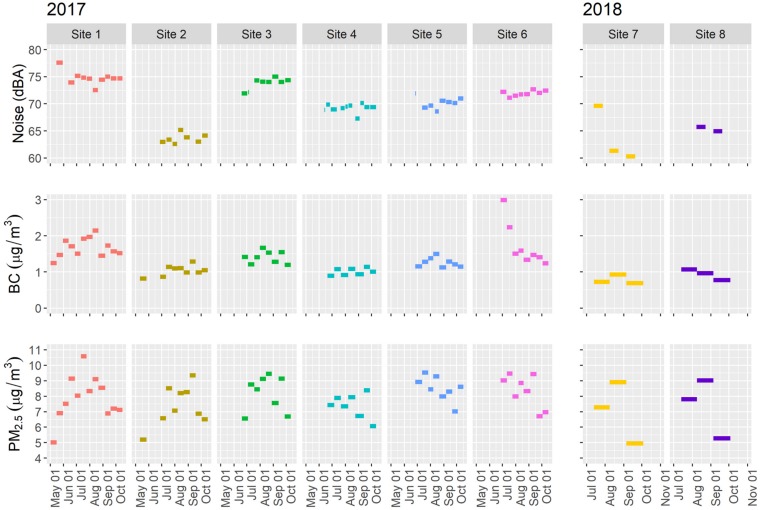
Time-integrated noise (top panel), black carbon (BC, middle panel), and particulate matter size 2.5 (PM_2.5_, bottom panel) measurements at the eight study sites. Measures are averaged over the period of times between site visits; approximately 2 weeks for Sites 1–6 and 4 weeks for Sites 7–8.

Measures of noise, BC, and PM_2.5_ before the opening of the online grocery delivery service warehouse are shown in Figure 2, averaged over the period of times between site visits; approximately 2 weeks for Sites 1–6 and 4 weeks for Sites 7–8. Noise varied substantially from 60.3 dBA to 77.6 dBA, with Sites 2 and 7 having generally lower noise levels than the other six sites. BC levels also demonstrated higher variability, ranging from 0.69 µg/m^3^ in September of 2018 at Site 7 to a high of 2.99 µg/m^3^ in late June at Site 6. PM_2.5_ ranged from 4.95 µg/m^3^ in September at Site 7 to 10.58 µg/m^3^ in mid-July, also at Site 1. The mean measured value for all sites and time periods was 7.88 µg/m^3^ (SD = 1.24) for PM_2.5_ and 1.33 µg/m^3^ (SD = 0.41) for BC. PM_2.5_ and BC were somewhat correlated at all sites, ranging from 0.40 to 0.89. 

In Table 2, we present the equivalent sound level for the entire measurement period and the median and inter-quartile range (IQR) for 15-min equivalent sound levels for each study site. Sites 1, 3, 5, and 6 exceeded the EPA’s recommended 70 dBA noise limit set to protect hearing. Daytime equivalent noise levels were generally higher than nighttime levels, except for Sites 4 and 6. Weekday equivalent noise levels were generally higher than weekend levels, except for Site 1.

Descriptive statistics for truck and total vehicle counts before and after the warehouse opened are shown in Table 3. Briefly, pre-opening 3-h window means ranged from a high of 268 trucks at Site 3 to a low of 6 trucks at Site 4. Mean vehicle count ranged from a high of 1446 at Site 3 to a low of 55 at Site 4. Post-opening means were in general slightly higher than pre-opening means, for both trucks and total vehicles. The time windows from 9 PM to midnight and midnight to 3 AM had the lowest traffic.

### 3.2. Objective 2: Modeled Changes in Traffic Flow

**Figure 3:**
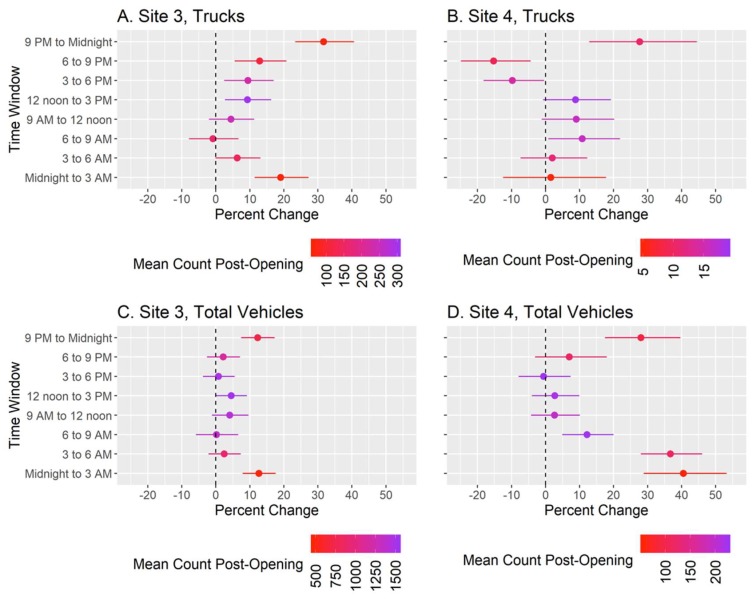
Percent change (points) and 95% confidence intervals (lines) in the truck flow at Site 3 (Panel A), truck flow at Site 4 (Panel B), total vehicle flow at Site 3 (Panel C), and total vehicle flow at Site 4 (Panel D) after the opening of an online grocery delivery service warehouse in a South Bronx neighborhood, compared to before the warehouse opened. Separate statistical models were completed for each three-hour time window; models controlled for day of the week and long-term and seasonal trends. Colors indicate the mean number of trucks or vehicles counted for a specific time window after the warehouse opened. Total vehicles include both cars and trucks.

Results from ITS models comparing traffic flow before and after the warehouse opened for Sites 3 and 4, by vehicle type and time window, are shown in Figure 3. We found statistically significant increases in truck flow for nearly all time windows for Site 3, with the highest occurring from 9 PM to midnight (Percent (%) Change = 31.7%, 95% CI = 23.4, 40.6%). The 9 PM to midnight window also showed the greatest increase for Site 4 (%Change = 27.7, 95% CI = 12.9, 44.5%), however, some time windows for this site also had significant decreases in truck flow. For total vehicles, Sites 3 and 4 each had significantly elevated total vehicle flow for the midnight to 3 AM (%Change = 12.7%, 95% CI = 7.9, 17.6% and 40.5%, 95% CI = 28.9, 53.3%, respectively) and 9 PM to midnight windows (%Change = 12.3%, 95% CI = 7.5, 17.3% and 28.1%, 95% CI = 17.5, 39.6%, respectively). In addition, the other two morning time windows for Site 4 also had significantly increased vehicle flow (6 to 9 AM and 3 to 6 AM windows). 

**Figure 4:**
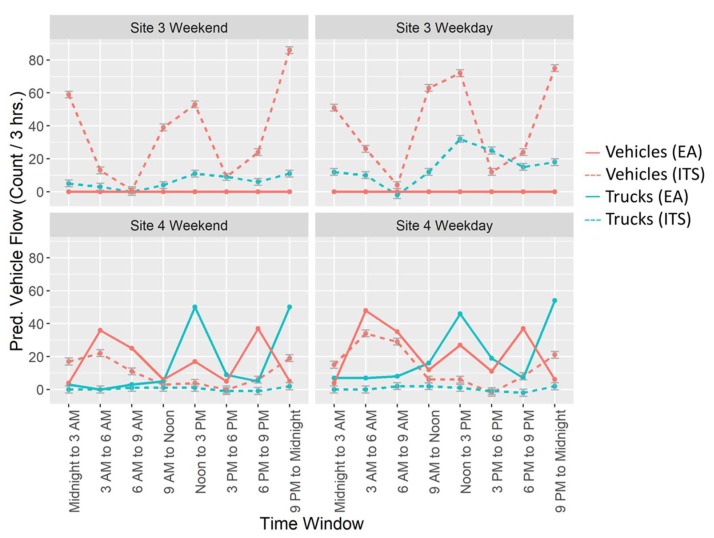
Predicted values for increases in truck (teal) and total vehicle (pink) flow due to the opening of an online grocery delivery service warehouse, from our interrupted time series (ITS) models (dashed lines) and the environmental assessment (EA) form submitted by the delivery service before construction (solid lines), separated by site and weekend vs. weekday. Gray error bars for the ITS points represent the mean +/− the standard error. Total vehicles include both cars and trucks.

Comparisons between the online grocery delivery service warehouse’s EA and predictions from the ITS model for Sites 3 and 4 are shown in Figure 4, separated by weekend/weekday, as done in the EA. The EA did not predict any additional vehicles or trucks traveling through Site 3, although the ITS models show substantial increases in flow for this site, for both weekends and weekdays. In contrast, the EA predictions in increased truck and vehicle flow for Site 4 are almost uniformly higher than those from the ITS models for both vehicles and trucks on weekends and weekdays. The only exceptions are for vehicle flow during the overnight time windows (9 PM to midnight and midnight to 3 AM), for which ITS models show greater total vehicle increases for both weekdays and weekends than predicted by the EA.

### 3.3. Objective 3: Increases in Traffic-Related BC and Noise

Results of the regression analyses used to estimate the contribution of segregated traffic flow *Q_car_* and *Q_tr_* to air and noise pollution are presented in Table 4. 

For Site 4, noise levels exhibited a significant dependence on the segregated traffic flows *Q_car_* and *Q_tr_*. The slope factor for trucks *λ_tr_* was about 13 times higher than that for cars, *λ_car_*, i.e., trucks contributed much more to noise levels than cars. The change in logarithmic sound-levels due to an additional 100 trucks per hour, $\partial L_{eq,15\min}/\partial Q_{tr}$, is also about 13 times higher than the corresponding change for cars, $\partial L_{eq,15\min}/\partial Q_{car}$. The slope factor for the total traffic flow, *λ_tot_*, lies between the one for cars and trucks. For Site 3, noise levels depended significantly on only one out of the two segregated traffic flows. 

**Figure 5:**
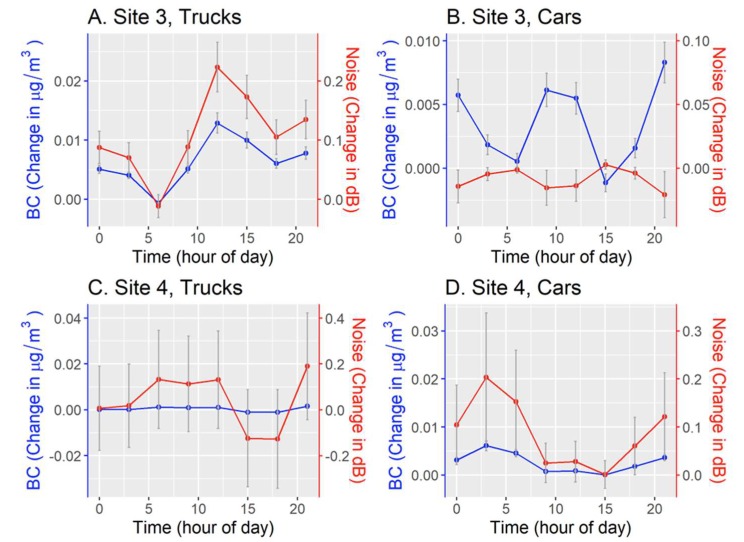
Estimated change in black carbon (blue lines) and noise (red lines) from the average count of trucks and cars attributed to the opening of an online grocery delivery service warehouse at two study sites. Points where the line segment changes direction corresponding to the value at the start hour of a 3-h time window (i.e., the value at time = 0 corresponds to the value at Midnight for the Midnight to 3 AM time window). Gray error bars represent the mean +/− the standard error.

Figure 5 shows our estimations of BC and noise generated by the additional truck and car traffic from the facility. Values are relatively low, with a mean change in BC of 0.003 µg/m^3^ (SD = 0.003) and 0.06 dBA (SD = 0.09) for noise. For Site 4, increases in noise were seen for early morning and late-night hours (from cars), whereas for Site 3 increases were highest around midday (from trucks). 

## 4. Discussion

### 4.1. Traffic Burden 

In a community already experiencing a substantial burden of air pollution, we found significantly increased truck and vehicle flow at both monitoring sites after the opening of the online grocery delivery service warehouse, particularly for overnight time windows, on the order of 10% to 40% change (see Figure 3). This increase in traffic translated to a mean predicted increase in BC of 0.003 µg/m^3^ and in noise of 0.06 dBA.

### 4.2. Environmental Injustice

While air pollution has been decreasing in NYC over time [5,6], we are concerned that specific communities, in particular those that already have higher than average amounts of pollutants from traffic and other sources, may benefit from this trend to a much smaller degree than others. Such communities are often comprised of people of color, have lower socio-economic status, and may have difficulty advocating for policy changes to support the health of their environment [40]. The Mott Haven neighborhood area is no exception to this trend; it has greater air pollution than both the Bronx borough and NYC, is predominantly Latino and of African descent, and has a high percentage of residents living in poverty [4]. In this case, the opening of a new distribution warehouse served to increase traffic, air pollution, and noise in a neighborhood already suffering from environmental injustice.

### 4.3. Black Carbon Burden

We estimated that the increase in truck and vehicle traffic resulted in a relatively small increase in BC. Prior studies have demonstrated an association between BC and asthma; a 2012 World Health Organization report on the health effects of BC found that a 10 µg/m^3^ increase in black smoke (~2.35 µg/m^3^ BC) [41] resulted in a 1.64% increase in asthma hospital admissions among children [42]. While the increase in black carbon we observed in the Mott Haven neighborhood was much less and may not translate to a measurable increase in asthma hospitalizations, the Mott Haven community already has some of the highest rates of childhood asthma emergency department visits in NYC [4]. 

### 4.4. Pre-existing Burdens

Important to consider in the decision to open a new warehouse or industrial facility in a neighborhood is the existing burden of negative health risk factors. Previous research suggests that the association between air pollution and health outcomes can be modified by socio-economic and race/ethnicity factors [1,2,3]. For example, a recent review found that risk for poor cardiovascular disease outcomes from air pollution was greatest among vulnerable populations, such as black individuals compared to whites, and those with low SES [2]. A study assessing the interaction of deprivation with NO_2_ and birth outcomes in NYC found an inverse association between birth weight and NO_2_ in the most deprived areas [3]. In addition, a study at the city level evaluating long-term PM_2.5_ exposure and mortality found increased risk of mortality from PM_2.5_ among cities with a greater percentage of the population living in poverty or without a high school diploma, and increased mortality among cities with a higher percentage of black inhabitants [1]. These risk factors are present in the Mott Haven neighborhood of the South Bronx, where pre-opening particulate pollution was already high and the community has lower SES and a high percentage of Hispanic and black residents [4]. Thus, we should be concerned that additional traffic might cause even worse health outcomes among the Mott Haven community than among residents of some other, more affluent areas. Rather than building facilities that increase traffic, a known risk factor for negative health outcomes including asthma, we advocate for the development of protective environmental structures such as public parks and open spaces [43]. Focus should be placed on reducing traffic exposure for neighborhoods similar to Mott Haven, rather than on allowing (even small) increases. 

### 4.5. Environmental Assessment

Despite the large size of the new warehouse and the expected substantial increases in traffic, an EIS specific to this development was not submitted. Instead, a less extensive EA form was submitted to the NYC Industrial Development Authority on behalf of the distribution warehouse [26]. This EA relied on an EIS created in 1993, 19 years prior to its submission [11], with some supplementation to provide a traffic analysis comparison with the original statement [26]. We have compared modeled increases in truck and vehicle traffic at two sites with those predicted by the EA. We found that for one site (Site 4), identified in the EA as the route for 35% of incoming traffic [26], modeled traffic flow was below that predicted by the EA for all time windows except for midnight to 3 AM. The second site (Site 3) was not identified on the EA as a route [26]; however, it had increased truck and vehicle traffic at overnight time windows as well. The assumption of no warehouse-related traffic at Site 3 seems unrealistic, because that site is at the last exit ramp that vehicles traveling Southbound on I-87 can take to reach the main entrance of the warehouse. Thus, the EA likely inadequately identified areas of increased traffic, as it entirely missed an important interstate exit at which we observed traffic increases (Site 3), which is next to a residential street [13]. Since we had traffic radar systems installed at only two sites, we were unable to examine the EA’s claim that total truck and vehicle increases caused by the warehouse in the entire street network would be similar to those predicted in the 1993 EIS. In addition, it is possible that as the warehouse grows and increases the number of trucks and vehicles needed to provide service, the currently observed increase in traffic will be exacerbated. 

### 4.6. Noise Burden

In addition to reduced air quality and potentially increased adverse health effects, traffic related pollution also includes increased noise. We estimate an average noise increase of 0.06 dBA from the increased traffic due to the online grocery delivery service warehouse, although this is an average over all time windows and sites, and thus does not adequately represent the annoyance caused by short durations of louder traffic noise, such as those caused by blown vehicle horns or truck air brakes. Perhaps our plots of percentage of vehicle flow change versus time of the day (Figure 3) provide a better means of assessing annoyance, e.g., between 9 pm and midnight, we predicted a 32% and 28% increase in the number of trucks for Sites 3 and 4, respectively.

### 4.7. Comparison to Other Noise Studies

The noise levels we measured in the South Bronx are higher than levels measured in certain smaller cities such as Cáceres, a medium-sized city in Spain [44], and eight cities in the UK [45]. Our noise results are, however, consistent with those of other studies conducted in NYC. For example, one study assessing noise levels from traffic found an average 10-min noise level *L_eq,_*_10 min_ of 69.3 dBA (+/− 4.1) [46]. A second study of 99 street sites in NYC found that the mean street noise level was 73.4 dBA, with substantial spatial variation (range 55.8-95.0 dBA) [47]. Our results are consistent with those studies; the equivalent sound levels *L_eq,tot_* for our eights sites ranged from 63.7 to 75.0 dBA. It should be noted that some of these averages are already over the 24-h exposure limit of 70 dB identified by the EPA as the safe margin to prevent measurable hearing loss [20], and thus further increases are undesirable. Another study of 56 sites in NYC [19] found that weekday noise levels were moderately higher than weekend levels (~2 dBA) which is consistent with our findings except for Site 1 where weekend levels were 0.3 dBA higher (see Table 2). That study also found daytime noise levels to be significantly higher than night levels, which is consistent with our study, except for Sites 4 and 6, but at these sites noise levels are only about 1 dBA higher.

Proximity to higher-traffic roads (as indicated by a lower value of the NYS code listed in Table 1) appears to be associated with higher noise levels. The four sites at which the 70 dBA limit was exceeded correspond to larger roads, as a comparison between the 5th and 6th column of Table 1 and the 3rd column of Table 2 shows. Our findings are consistent with a study in Guangzhou, one of China’s largest cities [48].

We compared regression coefficients of our traffic-noise model given by Equation (1) to those measured for the model proposed by Cannelli et al. [30] and Cocchi et al. [31]. The ratio of the contribution from trucks and cars to noise which we determined for Site 4, *λ_tr_* / *λ_car_ =* 12.6, can be compared to the ratio (denoted by *β* in their model) of 8 that Cannelli et al. [30] suggested for Italian roads. Our value is on the order of Canelli et al.’s, but higher. The difference could be due to differences in vehicle noise emission controls arising from geographic (Italy vs. the US) and temporal variation (Canelli et al. made their measurements 1979–1980). 

### 4.8. Other Traffic Burdens

While we have focused extensively on the air and noise pollution impacts of traffic, increased truck and car traffic may have many other negative outcomes for a community. These include reducing the ability to comfortably walk or cycle on local streets, the potential for increases in traffic accidents or pedestrian and cyclist accidents, increased travel times around the neighborhood or to local businesses, and decreased access to natural resources such as waterfronts or parks, because traffic can make it unsafe for community members to cross roads or find parking.

### 4.9. Limitations

This study has several limitations. First, air quality and noise were only measured before the warehouse opened, and not after. However, our estimates for warehouse-related BC level increases are based on mobile-source contributions to BC levels which we previously derived from actual BC and traffic measurements [13] as well as the measured warehouse-related changes in traffic; and similarly our predicted changes in noise levels are based on mobile-source contributions to noise levels we determined in this work from actual noise and traffic measurements as well as the measured warehouse-related changes in traffic. Second, it is possible that unmeasured confounding could impact our results and bias our estimates of traffic increase resulting from the warehouse opening, if some other variable also impacted traffic at the same point in time in the study area. For example, construction of the warehouse could have increased traffic before the facility opened, biasing our results towards the null. Third, we conservatively estimated the opening of the warehouse as October of 2018, however it is possible that the facility opened earlier. If this is the case, we likely underestimate the increase in traffic as a result of the facility opening. Fourth, the facility may increase traffic further in the future if their business continues to grow, making our estimations of the impact of the facility on traffic even further underestimated. Fifth, we use a limited number of sites to estimate changes at the neighborhood level, but these sites may not be representative of the entire neighborhood, although they were chosen to represent different street sizes and housing types. Sixth, due to limited participant availability we could not always change air sampling filters on exactly the same weekdays thereby hampering comparisons between sites due to different ratios of weekend/weekday traffic; however, we do not expect this to be a major limitation, because all time periods between visits for Sites 1 through 6 contained two weekends, and for Sites 7 and 8 four weekends. Finally, our study may not be generalizable to other neighborhoods or cities with different neighborhood characteristics and traffic patterns. 

## 5. Conclusions

In a community already differentially impacted by high levels of air pollution, we found that the opening of an online grocery delivery service warehouse significantly increased truck and vehicle flow, especially for overnight time windows, and that for one traffic monitoring site, resulting changes were not adequately predicted by the facility’s environmental assessment prior to construction. We estimate that these increases translate into small increases in black carbon and noise exposures for this neighborhood. However, even small increases are of concern because the community seeks to substantially decrease air pollution levels in their neighborhood, and incremental increases thwart their efforts to actuate such change. We suggest caution before building additional facilities in the area that may further increase traffic and its related pollution, as well as the submission of more thorough environmental assessments. Focus should be placed on decreasing traffic and pollution in overburdened communities, rather than incentivizing additional traffic-intensive facilities.

## Figures and Tables

**Table 1:** Characteristics of PM_2.5_ and noise monitoring locations.

Site		Distance from Curb [m]	Height [m]	Mounting Type	AADT ^†^	Road Class (NYS Code)
1		6 *	7	Window	10,013	Minor arterial (16)
2		6	3	Window	NA	Local road (19)
3		6	4	Window	~9000	Principal arterial interstate (11)
4	PM_2.5_	8	9	Flat roof	~1500	Local road (19)
	Noise	0	2	Light pole		
5		12	8	Window	6863	Minor arterial (16)
6		6	4	Window	24,991	Principal arterial other (14)
7		7	6	Flat roof	NA	Local road (19)
8		25	6	Flat roof	6863	Minor arterial (16)

^†^ AADT = Annual average daily traffic; NA = Not available; * Distance from motorized vehicle lanes, which are separated from the building through a bicycle lane and a sidewalk.

**Table 2:** Descriptive statistics of sound levels.

Site	*L_eq,_*_15min_ Median (IQR) (dBA)	*L_eq,tot_* (dBA)	*L_eq,daytime_* (dBA)	*L_eq,night_* (dBA)	*L_eq,weeeday_* (dBA)	*L_eq,weekend_* (dBA)	Number of Whole Days
1	73.3 (71.4, 75.1)	75.0	75.5	74.0	74.9	75.2	129
2	61.1 (59.0, 63.3)	63.7	64.5	61.9	64.3	61.8	90
3	73.7 (72.3, 74.9)	74.0	74.2	73.9	74.3	73.6	93
4	67.4 (65.2, 69.6)	69.3	69.0	69.8	69.5	67.4	93
5	68.7 (67.2, 70.4)	70.4	70.3	69.6	70.5	68.9	84
6	70.4 (68.8, 72.4)	72.0	71.5	72.7	72.5	70.6	103
7	59.1 (57.5, 61.7)	65.9	66.7	65.1	66.7	64.3	42
8	63.3 (62.2, 64.8)	65.4	65.9	64.5	65.6	65.1	28

**Table 3:** Descriptive statistics for measured truck and vehicle counts before and after the opening of an online grocery delivery service warehouse in a South Bronx neighborhood.

	Site 3 Mean Count (SD)		Site 4 Mean Count (SD)	
	Pre-Warehouse Opening	Post-Warehouse Opening	Pre-Warehouse Opening	Post-Warehouse Opening
**Trucks**				
Time Window				
Midnight to 3 AM	66.2 (28.1)	63.4 (23.9)	5.8 (3.1)	5.4 (3.5)
3 to 6 AM	126.8 (59.0)	137.8 (59.4)	11.4 (4.6)	10.7 (4.3)
6 to 9 AM	148.2 (68.0)	154.5 (67.3)	17.3 (8.7)	16.1 (8.5)
9 AM to 12 noon	217.8(102.4)	242.5 (109.4)	16.3 (7.8)	16.3 (7.9)
12 noon to 3 PM	268.3 (121.7)	303.6 (132.8)	17.0 (7.0)	19.3 (7.6)
3 to 6 PM	215.6 (96.9)	231.2 (102.3)	14.9 (6.2)	13.8 (5.7)
6 to 9 PM	104.6(43.4)	106.7 (45.1)	9.8 (4.3)	8.5 (4.0)
9 PM to Midnight	63.4(22.4)	65.1 (19.7)	7.6 (3.8)	10.4 (4.1)
**Vehicles**				
Time Window				
Midnight to 3 AM	492.7 (85.0)	470.6 (79.9)	55.0 (20.6)	54.6 (19.8)
3 to 6 AM	854.8 (282.1)	908.8 (266.4)	107.2 (36.5)	114.5 (34.8)
6 to 9 AM	1,294.4 (486.5)	1,354.0 (515.1)	215.5 (91.5)	224.9 (98.0)
9 AM to 12 noon	1,325.9 (363.5)	1,434.0 (390.7)	193.2 (71.9)	195.2 (76.8)
12 noon to 3 PM	1,445.7 (291.9)	1,538.8 (317.3)	202.7 (66.2)	216.6 (67.1)
3 to 6 PM	1,429.6 (258.7)	1,496.1 (264.8)	222.4 (87.0)	225.7 (79.9)
6 to 9 PM	1,078.1 (173.6)	1,139.6 (183.0)	126.8 (50.5)	116.1 (42.3)
9 PM to Midnight	728.6 (137.4)	710.7 (111.1)	81.8 (29.7)	93.3 (26.8)

Note: Pre-warehouse Opening time period = June 1, 2017 through September 30, 2018; Post-warehouse Opening time period = October 1, 2018 through May 5, 2019.

**Table 4:** Mobile source contributions to noise and black carbon. Confidence intervals are given in parentheses.

			**Noise**			
**Model**	*λ_tot_* [h]	$\frac{dL_{eq,15\min}}{dQ_{tot}}$ [dBA /(100 h^−1^)]	*λ_tr_* [h]	$\frac{\partial L_{eq,15\min}}{\partial Q_{tr}}$ [dBA / (100 h^−1^)]	*λ_car_* [h]	$\frac{\partial L_{eq}}{\partial Q_{car}}$ [dBA / (100 h^−1^)]
**Site 3**						
*Q_tr_ + Q_car_*	–	–	60,275 ^***^ (47,331, 73,219)	2.6	−1,679 (−5,161, 1,802)	−0.1
*Q_tot_*	9175 ^***^ (6,833, 11,518)	0.4	–	–	–	–
**Site 4**						
*Q_tr_ + Q_car_*	–	–	138,191 ^***^ (60,320, 216,063)	25.1	11,011 ^*^ (−335, 22,356)	2.0
*Q_tot_*	21,181 ^***^ (11,233, 31,129)	3.8	–	–	–	–
**Black Carbon**						
ln(BC)-traffic model			*α*_tr_ [μg/m^3^ per 100 trucks/h]		*α*_car_ [μg/m^3^ per 100 cars/h]	
**Site 3**						
$\begin{array}{l} \;Q_{tr}+\;Q_{car}+\\ \;{\bar{Q}}_{tot}\;\mathrm{RS} \end{array}$	–	–	0.15 *** (0.13, 0.18)	–	0.04 *** (0.03, 0.05)	–
**Site 4**						
$\begin{array}{l} \;Q_{tr}+\;Q_{car}+\\ \;{\bar{Q}}_{tot}\;\mathrm{RS} \end{array}$	–	–	0.21 *** (0.10, 0.31)	–	0.06 *** (0.03, 0.08)	–

Note: * *p* < 0.05; *** *p* < 0.001.

## Abbreviations

BC	Black carbon
EA	Environmental assessment
EIS	Environmental impact statement
ITS	Interrupted time series
NYC	New York City
PM_2.5_	Particles with aerodynamic diameter < 2.5 μm
SBU	South Bronx Unite
SES	Socio-economic status
